# Coinfections in Tuberculosis in Low- and Middle-Income Countries: Epidemiology, Clinical Implications, Diagnostic Challenges, and Management Strategies—A Narrative Review

**DOI:** 10.3390/jcm14072154

**Published:** 2025-03-21

**Authors:** Ramona Cioboata, Mara Amalia Balteanu, Andrei Osman, Silviu Gabriel Vlasceanu, Ovidiu Mircea Zlatian, Denisa Maria Mitroi, Oana Maria Catana, Adriana Socaci, Eugen-Nicolae Tieranu

**Affiliations:** 1 Pneumology Department, University of Medicine and Pharmacy, 200349 Craiova, Romania; ramona_cioboata@yahoo.com; 2 Department of Pulmonology, Faculty of Medicine, Titu Maiorescu University, 031593 Bucharest, Romania; mara.balteanu@prof.utm.ro; 3 Department of Anatomy and Embryology, University of Medicine and Pharmacy, 200349 Craiova, Romania; 4 Department of Physiology, “Carol Davila” University of Medicine and Pharmacy, 050474 Bucharest, Romania; 5 Department of Microbiology, University of Medicine and Pharmacy, 200349 Craiova, Romania; ovidiu.zlatian@umfcv.ro; 6 Doctoral School, University of Medicine and Pharmacy, 200349 Craiova, Romania; denisa_maria2@yahhoo.com (D.M.M.); oana_cattana@yahoo.com (O.M.C.); 7 Department of Biology and Life Science, Vasile Goldis University Arad, 310025 Arad, Romania; socaci.adriana@uvvg.ro; 8 Department of Internal Medicine-Cardiology, University of Medicine and Pharmacy, 200349 Craiova, Romania; eugen.tieranu@umfcv.ro

**Keywords:** tuberculosis, coinfections, HIV, COVID-19, low- and middle-income countries

## Abstract

Tuberculosis (TB) continues to be a major public health challenge in low- and middle-income countries (LMICs), where high burdens of coinfections exacerbate the disease’s impact. In 2023, an estimated 8.2 million people were newly diagnosed with tuberculosis worldwide, reflecting an increase from 7.5 million in 2022 and 7.1 million in 2019. In LMICs, limited access to healthcare, inadequate nutrition, and poor living conditions contribute to higher coinfection rates among TB patients, leading to delayed diagnosis and treatment, which in turn exacerbates disease severity and facilitates transmission. This narrative review synthesizes the epidemiology, clinical implications, diagnostic challenges, and management strategies related to TB coinfections with viral pathogens including HIV, SARS-CoV-2, and influenza, bacteria such as *Streptococcus pneumoniae*, *Staphylococcus aureus*, *Klebsiella pneumoniae*, and *Pseudomonas aeruginosa*, fungi such as *Aspergillus* and *Candida* species, and parasites. This review highlights that overlapping symptoms, immune system compromise, and socioeconomic barriers in LMICs lead to delayed diagnoses and suboptimal treatment outcomes, while also addressing the challenges of managing drug interactions particularly in HIV–TB coinfections and underscoring the need for integrated diagnostic approaches, improved treatment regimens, and strengthened healthcare systems, thereby consolidating current evidence to inform future research priorities and policy interventions aimed at reducing the overall burden of TB and its coinfections in resource-limited settings.

## 1. Introduction

Tuberculosis (TB) remains a significant global health challenge, particularly in low- and middle-income countries where the burden of coinfections is substantial. In 2023, an estimated 8.2 million people were newly diagnosed with tuberculosis worldwide, marking an increase from 7.5 million in 2022 and 7.1 million in 2019. These figures were significantly higher than the 5.8 million and 6.4 million cases reported in 2020 and 2021, respectively [1]. The surge in cases during 2022 and 2023 likely reflects a backlog of individuals who contracted tuberculosis in earlier years but faced delays in diagnosis and treatment due to disruptions caused by the COVID-19 pandemic [2].

The highest incidence occurred in the South-East Asia and African regions, with the African region alone accounting for approximately 2.55 million cases (95% UI: 2.25–2.87 million), equivalent to an incidence rate of 206 per 100,000 population. In contrast, the European and the Americas regions reported significantly lower incidence rates, at 24 and 33 cases per 100,000 population, respectively. The concentration of cases was most pronounced in high TB burden countries, which collectively reported approximately 9.41 million cases (95% UI: 8.62–10.2 million), underscoring the disproportionate impact of TB in low- and middle-income countries compared to high-income regions [3].

The incidence of tuberculosis in high-income countries is generally quite low, often below 10 cases per 100,000 population. In contrast, many low- and middle-income countries (LMICs) experience incidence rates that can exceed 200 cases per 100,000 population. This means that, in some settings, the burden of TB in LMICs can be roughly 20 to 30 times higher than in high-income countries. Additionally, LMICs account for over 95% of global TB cases and deaths, underscoring a substantial disparity in disease burden between these regions and high-income settings [1,3].

The 2024 report indicates that over 25% of the world’s population is estimated to carry TB infection referred to as latent TB infection (LTBI), which remains asymptomatic with about 5% of those individuals progressing to active TB disease within the first two years after infection [4]. LTBI is characterized by the presence of *Mycobacterium tuberculosis* in the body without any symptoms, radiological abnormalities, or microbiological evidence of active disease, and because it is not contagious, it does not contribute to the spread of TB. In contrast, active TB is a clinical condition in which the infection manifests with symptoms and is confirmed through radiological and microbiological tests; active TB is contagious and can spread to others via respiratory droplets [5].

TB was responsible for approximately 1.25 million deaths in 2023 (95% UI: 1.13–1.37 million), with 1.09 million occurring among HIV-negative individuals and 161.000 among those with human immunodeficiency virus (HIV). The “95% UI” (95% uncertainty interval) indicates that there is a 95% probability that the true number of TB deaths falls between 1.13 and 1.37 million, as estimated using Bayesian methods. Notably, TB deaths among HIV-negative individuals were nearly double the 0.63 million deaths attributed to HIV/AIDS (Acquired Immunodeficiency Syndrome). Moreover, TB mortality was significantly more impacted by the COVID-19 pandemic than HIV/AIDS [3]. In low- and middle-income countries, the burden of TB is further compounded by the high costs and limited access to healthcare [6,7]. The financial burden on patients and healthcare systems is significant, particularly when dealing with TB multimorbidity, which includes chronic conditions like diabetes and HIV. The prevalence and risk of chronic conditions in TB patients are higher in these regions, leading to poorer health outcomes and increased disease burden [8].

Furthermore, emerging evidence suggests that infections can lead to persistent immunological sequelae. For instance, a recent multinational cohort study demonstrated an increased risk of incident allergic diseases following COVID-19 infection, highlighting the potential for long-term immune dysregulation post-infection. This finding underscores the need for integrated diagnostic and therapeutic strategies in managing coinfections, including TB, in resource-limited settings [9].

The immune response in TB patients is often compromised, making them more susceptible to secondary infections [10]. This heightened vulnerability arises from a complex interplay of immune system alterations and pathogen interactions. In particular, pulmonary TB frequently facilitates secondary bacterial and fungal infections, which obstruct alveolar airflow and promote tissue symbiosis [11]. These pathological changes contribute to the development of chronic obstructive lung disease, significantly increasing morbidity and mortality rates [12]. Such complications underscore the critical need for targeted interventions to mitigate the impact of secondary infections in TB management and improve patient outcomes [13].

In HIV patients, superinfection with TB significantly complicates clinical management. In contrast, coinfections in TB patients, such as, bacterial infections like pneumonia, fungal infections such as *Aspergillus*, viral infections like influenza and hepatitis, and parasitic infections like helminths, complicate the clinical management and progression of TB [14,15,16,17]. These coinfections can adversely affect treatment outcomes and immune responses, necessitating a comprehensive understanding of their epidemiology, diagnostic challenges, and management strategies. Understanding TB coinfections is crucial, particularly given the disease’s recurrence [18].

Overall, the global burden of TB is a complex interplay of various factors, including coinfections, healthcare access, and socioeconomic conditions. Addressing these challenges requires a multifaceted approach that includes improved diagnostic techniques, effective treatment strategies, and comprehensive healthcare policies to reduce the disease’s impact on affected populations.

In this context, this manuscript follows a narrative review approach to synthesize current evidence on TB coinfections in LMICs by integrating relevant findings from clinical studies, observational reports, and expert consensus published primarily over the past two decades. Specifically, we examine epidemiological trends, clinical implications, diagnostic challenges, and management strategies related to key coinfections, including viral pathogens, significant bacterial pathogens, major fungal species, and prevalent parasitic infections. By critically evaluating evidence from multiple sources, identifying gaps in knowledge, and assessing their impact on TB progression and treatment outcomes, we aim to provide insights that inform future research and enhance TB coinfection management in resource-constrained settings.

## 2. Viral Coinfections

Tuberculosis and viral coinfection present a significant public health challenge, particularly in regions with high burdens of both diseases. This review centers on viral coinfections that have the most substantial impact on TB management in LMICs.

Specifically, we discuss coinfections with HIV, SARS-CoV-2, and influenza, as these viruses are not only highly prevalent but also critically influence TB progression and treatment outcomes. This targeted selection allows us to synthesize evidence on the interplay between TB and these key viral pathogens, which are central to current public health challenges.

### 2.1. Influenza Coinfection

Coinfection with influenza and TB is associated with increased mortality, although the magnitude of this risk differs depending on the patient population evaluated. In one study, among patients with laboratory-confirmed tuberculosis, those coinfected with influenza had an approximately 2.3-fold higher risk of death (adjusted Odds Ratio [aOR], 2.3; 95% UI, 1.02–5.2). In contrast, when the analysis was restricted to patients with confirmed influenza, the presence of tuberculosis was associated with an even greater risk of death (aOR, 4.5; 95% UI, 1.5–13.3). These differences arise because the aORs were calculated in two distinct subgroups TB patients and influenza patients each with its own baseline risk profile and clinical characteristics [19]. Influenza infection can impair the control of *Mycobacterium tuberculosis* (Mtb), leading to enhanced bacterial growth and decreased survival in coinfected individuals. This is mediated through a type I interferon receptor-dependent pathway, which exacerbates TB progression [20,21]. Influenza virus coinfection can significantly alter the host’s immune response to TB. It has been shown to suppress Mtb-specific IFN-γ production, leading to increased bacterial loads and hyperinflammation in the lungs. This is primarily due to type I IFN signaling, which impedes the pulmonary migration of Mtb-specific CD4+ T cells [22]. Additionally, type I interferon signaling, induced by influenza, has been shown to enhance mycobacterial growth and impede the pulmonary influx of Mtb-specific Th1 cells, further exacerbating TB immunopathogenesis [21]. Coinfection results in differential cytokine profiles, with increased levels of IFN-γ+IL-17+CD4+ and IFN-γ+IL-17-CD8+ cells, and altered levels of cytokines such as IL-17A and MCP-1, which are associated with higher bacterial loads in TB patients [22]. The IL-10 signaling pathway plays a crucial role in enhancing susceptibility to Mtb during concurrent influenza infection. Blocking IL-10 receptor signaling has been shown to reduce bacterial loads in coinfected mice to levels comparable to those in Mtb only infected animals, suggesting a potential therapeutic target for managing coinfections [20].

Diagnosing coinfection can be challenging due to overlapping symptoms and the potential for influenza to mask TB symptoms. In some cases, TB may be underdiagnosed in patients presenting with influenza-like symptoms, particularly during influenza epidemics [23]. The interaction between influenza and TB complicates the clinical management of both diseases. The increased bacterial load and altered immune responses in coinfected patients suggest a need for targeted therapeutic strategies, including the potential benefit of influenza vaccination in TB-endemic settings [20,21].

For patients with TB and influenza coinfection, an integrated treatment approach is essential. Oseltamivir and zanamivir, which are effective against both influenza A and B viruses, are recommended for high-risk individuals and can reduce the duration of influenza symptoms by about one day, although their effectiveness is limited if initiated late and there is a potential for resistance [24]. Concurrently, the standard treatment for drug-sensitive tuberculosis involves a strict 6-month regimen of isoniazid, rifampin, pyrazinamide, and ethambutol to prevent relapse and the development of drug resistance [4]. These approaches aim to improve patient outcomes and address the challenges posed by drug resistance.

### 2.2. HIV Coinfection

HIV is one of the most critical viral coinfections in TB patients, significantly increasing the risk of developing active TB due to the immunosuppressive nature of the virus [3]. HIV and tuberculosis coinfection present a significant global health challenge, particularly in resource-limited settings. The relative risk of TB among HIV-infected individuals is significantly higher compared to those uninfected, with a 20- to 37-fold increase depending on the HIV epidemic’s state [25].

The prevalence of HIV/TB superinfection varies significantly by region, with particularly high rates observed in sub-Saharan Africa, where the dual burden of both infections remains a major public health challenge [26]. In some settings, up to 70.5% of TB cases are HIV-positive, underscoring the strong epidemiological link between the two diseases [27]. This is due to the immunosuppressive nature of HIV, which accelerates the progression from latent to active TB and vice versa. While elevated HIV/TB superinfection rates have also been reported in some high-income countries, such as the United States and Russia, the underlying factors in these contexts differ. In non-LMIC settings, coinfection dynamics are influenced by socioeconomic factors, healthcare access disparities, and risk behaviors such as injection drug use. However, in LMICs, additional challenges such as limited diagnostic capacity, under-resourced healthcare systems, and higher prevalence of comorbid conditions further exacerbate the burden of TB/HIV coinfection [28,29]. This regional variation highlights the need for targeted interventions and integrated TB/HIV management strategies that are specifically tailored to the resource constraints and unique challenges of LMIC settings [30].

The COVID-19 pandemic has significantly disrupted healthcare services for HIV and TB in LMICs. Studies indicate that the pandemic could lead to increased mortality rates for these diseases due to interruptions in antiretroviral therapy for HIV and delays in TB diagnosis and treatment [31,32]. These disruptions could result in a substantial loss of life-years, comparable to the direct impact of COVID-19 itself in high-burden settings [33]. Healthcare access for HIV and TB patients in LMICs has been severely impacted by the pandemic. Barriers such as fear of COVID-19 disease, transport disruptions, and movement restrictions have made it difficult for patients to reach healthcare facilities. Additionally, non-medical support services, like food supplementation and counseling, have become harder to access, further complicating patient care [31]. The interaction between TB and HIV exacerbates disease progression and severity, leading to increased morbidity and mortality. This comorbidity complicates clinical presentation and management, often resulting in poorer treatment outcomes and higher fatality rates [17,34]. Pulmonary tuberculosis remains the most common manifestation among patients coinfected with HIV, but extrapulmonary TB (EPTB) is also a significant concern in this population. In particular, forms such as miliary TB, TB meningitis, and lymph node involvement are more frequently seen among HIV-infected individuals due to their compromised immune systems [27,35]. Moreover, unique clinical presentations can arise in these patients, including ocular TB manifesting as anterior uveitis or chorioretinitis and oral TB, which may appear as non-healing oral ulcers and, in some cases, serves as the first detectable indicator of HIV infection [36,37]. The spectrum of EPTB in HIV-infected persons often spans beyond these presentations and can include skeletal, genitourinary, and abdominal TB, underscoring the need for a broad clinical suspicion [38]. HIV infection increases the likelihood of atypical and disseminated TB presentations, including smear-negative pulmonary TB and higher rates of extrapulmonary disease, making diagnosis more challenging [29]. Common symptoms include weight loss, dyspnea, fever, night sweats, and cough. Conventional smear microscopy often fails to detect TB in HIV-infected individuals, necessitating the use of nucleic acid amplification tests for rapid and accurate diagnosis, especially in smear-negative cases [26]. These factors often delay diagnosis and complicate treatment initiation. Standard diagnostic tests like tuberculin skin tests (TSTs) and interferon gamma release assays (IGRAs) are less effective in active TB cases among HIV patients, requiring alternative diagnostic approaches [39]. However, advancements in diagnostic techniques, such as nucleic acid amplification tests like the Xpert MTB/RIF assay, have significantly improved the ability to detect TB in this population, facilitating earlier and more accurate diagnoses [40].

HIV-infected patients generally respond well to the standard 6-month antituberculosis treatment regimens, although mortality rates remain high due to complications such as drug interactions and adverse reactions [41]. A study comparing 6-month and 9-month regimens found that while both regimens had similar treatment success rates, the 9-month regimen resulted in a lower bacteriological recurrence rate [42]. The standard 6-month anti-TB regimen (rifampin, isoniazid, pyrazinamide, ethambutol) remains effective for patients coinfected with HIV; however, the rate of relapse can be higher in HIV-infected patients compared to those without HIV. Initiating antiretroviral therapy (ART) during TB treatment is crucial for improving survival rates among coinfected individuals [43]. However, the timing must be carefully considered based on the patient’s immune status. Early initiation in those with severe immunosuppression can reduce mortality but also increases the risk of immune reconstitution inflammatory syndrome (IRIS), a potentially severe inflammatory response triggered by immune recovery [44]. Thus, an individualized approach to treatment timing is necessary to optimize outcomes while minimizing complications. However, the administration of ART and anti-TB drugs can lead to significant drug interactions that affect treatment efficacy and safety exemplified by the Cytochrome P450 3A4 (CYP3A4) metabolized bedaquiline [45] (whose exposure is significantly increased by lopinavir/ritonavir but not by nevirapine), rifampicin’s potent induction of CYP3A4 (leading to subtherapeutic levels of protease inhibitors and prompting the use of rifabutin instead), and the need for dose adjustments when high-dose rifampicin alters the pharmacokinetics of efavirenz and dolutegravir [46,47].

Effective management of HIV and TB superinfection requires a comprehensive strategy that addresses the distinct clinical manifestations, diagnostic complexities, and overlapping treatment concerns posed by these intertwined diseases [17]. Prompt detection is essential, followed by meticulous coordination of antitubercular and antiretroviral therapies to reduce drug interactions and overlapping toxicities [48]. Close monitoring for complications such as immune reconstitution inflammatory syndrome is also essential to prevent exacerbation of adverse effects. Ultimately, achieving a balance between efficacy and safety ensures both infections are adequately treated, reducing morbidity and mortality while curbing further transmission [26].

### 2.3. SARS-CoV-2 Coinfection

**Figure 1:**
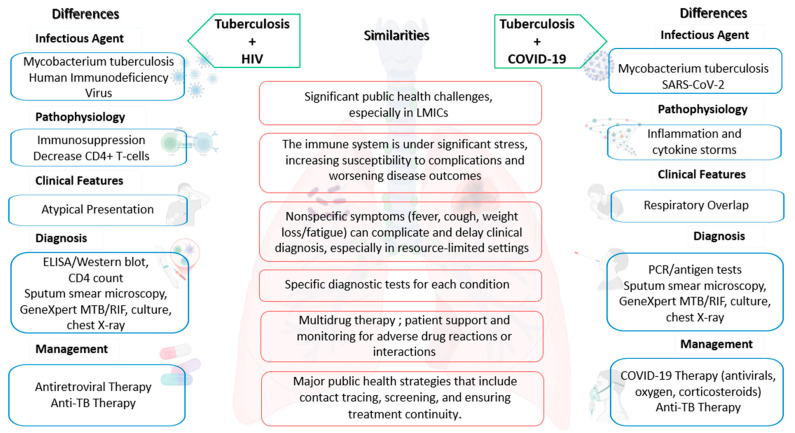
The similarities and differences between HIV/TB superinfection and TB/COVID-19 coinfection; LMICs—low- and middle-income countries; TB—tuberculosis; PCR—polymerase chain reaction; HIV—human immunodeficiency virus.

Tuberculosis and COVID-19, caused by SARS-CoV-2, are significant global health challenges, particularly in LMICs. The coinfection of these diseases poses unique challenges due to their overlapping symptoms and the compounded impact on the immune system, similar to the challenges encountered in HIV and TB superinfection (Figure 1).

TB and COVID-19 coinfections have been reported globally, with varying prevalence rates. In South Africa, the prevalence among specific populations ranges from 0.04% to 0.06%, and these coinfections pose a higher fatality risk than COVID-19 alone. Overall, TB and COVID-19 coinfection is a significant concern in LMICs such as India, the Philippines, and South Africa, where in-hospital fatality rates can reach up to 22.5% markedly higher than in high-income countries [49]. Coinfected patients face higher mortality rates, with studies indicating a mean fatality rate of 13.9% for TB-COVID coinfections. The mortality rate is higher in LMICs compared to high-income countries [49]. Furthermore, the COVID-19 pandemic has disrupted TB control efforts, exacerbating the public health burden in these regions [50]. Patients with TB and COVID-19 coinfection often exhibit overlapping respiratory symptoms, such as fever, cough, and respiratory distress, which complicates the clinical picture and can lead to misdiagnosis [51,52]. The presence of comorbidities [49] like smoking, diabetes, and hypertension is common among these patients, further complicating their clinical management [53,54,55]. Coinfected patients may also show higher levels of biomarkers such as C-reactive protein and ferritin, which can aid in suspecting TB in COVID-19 patients and vice versa [54,56]. The similarity in respiratory symptoms between tuberculosis and COVID-19 can lead to significant diagnostic delays, especially in LMICs where healthcare resources are limited [54]. Radiological findings such as upper lobe involvement and pleural effusion tend to be more common in coinfected patients and can help differentiate them from those with only COVID-19 [57]. To prevent disease spread and worsening outcomes, routine screening for TB in COVID-19 patients is recommended, particularly in high TB burden areas. Accurate detection relies on the combined use of diagnostic tools, including GeneXpert Ultra for TB and real-time reverse transcriptase–polymerase chain reaction (RT-PCR) for COVID-19 [33].

In addition to conventional diagnostic methods, advanced machine learning techniques are increasingly being applied to enhance patient detection. For instance, an adaptive ensemble deep learning framework has been developed for the reliable detection of pandemic patients, achieving an accuracy of 98.32% in identifying COVID-19 cases from hospital surveillance images [58,59,60]. This innovative approach exemplifies how deep learning can improve rapid and accurate diagnosis, a strategy that could be adapted to bolster the detection of TB coinfections in LMICs.

TB and COVID-19 coinfection can lead to a dysregulated immune response; patients with active TB and COVID-19 commonly exhibit decreased total lymphocyte counts and impaired CD4 T-cell responses, potentially exacerbating disease severity. Conversely, latent TB infection may modulate immune responses positively, although the evidence for this effect remains limited [61]. Treatment regimens for TB and COVID-19-coinfected patients are highly heterogeneous, often incorporating a combination of standard anti-TB drugs, antibiotics, antiviral therapies, and, in some cases, adjunct treatments such as hydroxychloroquine or lopinavir/ritonavir [62]. In LMICs, these regimens typically follow national COVID-19 guidelines alongside anti-TB therapy, although the efficacy of certain treatments including hydroxychloroquine and lopinavir/ritonavir remains variable [33,54,62]. The management of coinfection requires a multidisciplinary approach to ensure effective treatment and minimize adverse outcomes. Overall, effective management of TB and COVID-19 coinfection requires a multidisciplinary approach to ensure optimal treatment outcomes and minimize adverse events [63].

TB and COVID-19 coinfection pose a significant threat, particularly in LMICs, due to diagnostic challenges and increased mortality risk. Effective management requires integrated diagnostic and treatment approaches, considering the unique immune responses and comorbidities associated with coinfection.

## 3. Bacterial Coinfections

Bacterial coinfections are a significant concern in tuberculosis patients, complicating both the progression and treatment of the disease. These coinfections can lead to increased morbidity and complicate the clinical management of TB. We have chosen to focus on pathogens such as *Streptococcus pneumoniae*, *Staphylococcus aureus*, *Klebsiella pneumoniae*, *Pseudomonas aeruginosa*, and *Clostridium difficile* due to their high incidence, potential for multidrug resistance, and the complications they introduce in TB treatment [64,65,66,67]. These bacteria not only increase the clinical burden of TB but also pose diagnostic and therapeutic challenges, making them highly relevant for understanding and managing coinfections in LMICs.

*Klebsiella pneumoniae* and *Pseudomonas aeruginosa* also pose substantial challenges, particularly in immunocompromised TB patients and in healthcare settings with limited diagnostic capacity [66]. Coinfections with TB and other bacterial pathogens are prevalent in LMICs, where healthcare resources may be limited. For instance, *Streptococcus pneumoniae* is a leading cause of community-acquired pneumonia in Africa, with a prevalence of 21.4% among presumptive TB cases in Ethiopia, and it shows high levels of multidrug resistance [68]. Similarly, *Klebsiella pneumoniae* is a common nosocomial pathogen, particularly in immunocompromised patients such as those coinfected with HIV and TB, as observed in Mali [66]. *Staphylococcus aureus* is also frequently isolated in pediatric bloodstream infections in Africa, with a significant portion being methicillin-resistant [69].

Coinfection with *Streptococcus pneumoniae* and *Mycobacterium tuberculosis* has been documented primarily in HIV-seropositive patients. These patients are more susceptible to pulmonary infections with multiple organisms, including these two pathogens, due to their compromised immune systems [65,70]. In regions with high TB prevalence, such as parts of Africa and Asia, the likelihood of encountering such coinfections increases, although it remains uncommon in the United States [65]. In a high HIV/TB prevalence setting, a post hoc analysis of pulmonary tuberculosis among 39,836 participants in a phase III PCV9 trial in South Africa demonstrated a 43.4% (95% CI, 9.7–65.1%) overall reduction in culture-confirmed pulmonary tuberculosis hospitalizations (47.3% [95% CI, 8.6–69.6%] in HIV-infected children) often presenting with acute pneumonia resolving on empiric antibiotics suggesting superimposed pneumococcal infection and underscoring the need to investigate underlying TB [71].

Coinfections with *Mycobacterium tuberculosis* often present with symptoms typical of community-acquired pneumonia (CAP) such as cough, chest pain, and shortness of breath but the presence of TB can obscure or delay diagnosis and treatment if not recognized early [65,70]. In children, particularly those with HIV, these infections may initially respond to empiric antibiotics for acute pneumonia before revealing underlying TB. Rare instances of *Staphylococcus aureus* and TB coinfection have been described, sometimes manifesting as retropharyngeal abscesses that compromise the airway in infants [72]. Similarly, TB involving the cervical vertebrae with accompanying retropharyngeal and parapharyngeal abscesses has been reported in adults, underscoring the diagnostic complexity of such atypical presentations [73]. Coinfection with *Klebsiella pneumoniae* and *Mycobacterium tuberculosis* is a major clinical challenge that produces overlapping respiratory symptoms, especially in individuals with comorbidities such as diabetes, hypertension, and cardiovascular disease. This overlap often leads to delayed diagnosis and inadequate treatment, contributing to high mortality rates. Patients with severe pulmonary tuberculosis and concurrent *Klebsiella pneumoniae* infection typically exhibit compromised immune systems, lower CD8 counts, and poor nutritional status; elderly patients with multiple comorbidities are particularly vulnerable.

Microbiologically, *Klebsiella pneumoniae* strains in coinfection cases frequently display multidrug resistance, including extended-spectrum β-lactamase (ESBL) production and carbapenem resistance. Common strain types include ST23, ST15, and ST273, with a notable proportion being hypervirulent K1/K2 strains [15]. Nosocomial infections further complicate the clinical picture, especially among immunocompromised patients, such as those with HIV, where these infections may be mistaken for poor responses to tuberculosis treatment [15,66,74].

TB combined with other pathogens, such as *Pseudomonas aeruginosa*, may lead to significant immune suppression, partly through activation of the tryptophan-kynurenine pathway, which promotes an immunosuppressive environment conducive to pathogen persistence [75]. The coexistence of *Pseudomonas aeruginosa* in patients with non-tuberculous mycobacteria (NTM) infections can exacerbate metabolic disruptions and niche specialization, fostering chronic lung colonization [75].

A study reported that 10.91% of microbiologically confirmed pulmonary TB patients had significant bacterial coinfections, with *Pseudomonas aeruginosa* emerging as the most common pathogen present in 36.84% of these cases followed by *Acinetobacter baumannii* and *Klebsiella pneumoniae* [76]. Another study further highlighted the prevalence of *Pseudomonas aeruginosa* in TB-suspected patients by identifying it in sputum samples, underscoring its notable presence in such settings [77].

The detection of secretory immunoglobulin A (sIgA) in saliva has been proposed as a useful diagnostic marker, reflecting an immune response that may facilitate early detection and management of Pseudomonas infection in TB patients. Moreover, in patients with presumptive TB who ultimately test negative, *Pseudomonas aeruginosa* remains a significant pathogen in lower respiratory tract infections (LRTIs). Although 16S rRNA sequencing is capable of identifying *Pseudomonas aeruginosa*, its limitations in effectively managing patients with negative TB tests highlight the need for more advanced diagnostic tools [78].

Tuberculosis patients have an elevated risk of *Clostridium difficile* infection due to factors such as broad-spectrum antibiotic use, proton pump inhibitors, comorbidities, older age, and prolonged hospitalization, which heighten susceptibility in nosocomial settings [67]. Consequently, thorough diagnostic evaluations and tailored, multidrug treatment strategies are essential for managing these complex coinfections and improving patient outcomes [79]. Managing TB bacterial coinfections is challenging due to overlapping clinical presentations and the necessity for a high index of suspicion especially when another pathogen, such as *Streptococcus pneumoniae*, initially appears to explain a patient’s symptoms. Delays in recognizing underlying TB can lead to inappropriate treatment and isolation measures, worsening patient outcomes. In high-prevalence settings for HIV and TB, healthcare providers should maintain vigilance for TB even when community-acquired pneumonia pathogens are detected. Among HIV-infected children, pneumococcal vaccination has shown promise in reducing culture-confirmed TB, suggesting a preventive role against superimposed infections [65,70]. Coinfections involving *Staphylococcus aureus* and *Mycobacterium tuberculosis* can present with rare and complex symptoms, such as retropharyngeal abscesses requiring advanced imaging and molecular tests for accurate diagnosis [73]. Likewise, *Pseudomonas aeruginosa* and TB coinfections demand specialized diagnostic approaches: bronchoalveolar lavage can help detect both pathogens in patients with severe pneumonia [80,81], while emerging technologies such as electronic nose systems offer rapid and automated differentiation of *Mycobacterium species* from other lung pathogens [82]. Targeting disulfide bond forming enzymes, such as DsbB (a key component of the disulfide bond pathway) in *Pseudomonas aeruginosa* and VKOR (vitamin K epoxide reductase) in *Mycobacterium tuberculosis*, represents a novel approach to developing antibiotics or anti-virulence agents. These enzymes are crucial for bacterial pathogenicity and survival, making them attractive targets for drug development [83]. Additionally, novel antimicrobial agents, such as analogs of the frog-skin peptide Temporin 1 Tb, show promise in combating biofilm-mediated infections by *Pseudomonas aeruginosa*, potentially offering new therapeutic avenues [84].

Studies indicate that tuberculosis patients face an elevated risk of developing *Clostridium difficile* infection (CDI). The reported incidence varies across different settings: one study in a South African TB hospital found approximately 70.07 cases per 1000 patient admissions, with a high prevalence of multidrug-resistant *C. difficile* strains. Another study noted an incidence of 2 cases per 1000 adults receiving TB medication, highlighting that CDI is not uncommon in this population [85]. *Clostridium difficile* infection in tuberculosis patients can be difficult to diagnose due to overlapping gastrointestinal symptoms and the presence of multidrug-resistant strains, such as ribotype 017 in TB hospitals. Ribotype 017 is a highly virulent *C. difficile* strain that lacks the toxin A gene (tcdA-negative) but produces toxin B (tcdB-positive), contributing to severe infections, high relapse rates, and resistance to fluoroquinolones, which complicates treatment in TB patients undergoing prolonged antibiotic therapy. A two-step diagnostic algorithm, often incorporating nucleic acid amplification tests, is used to differentiate true infection from asymptomatic carriage [86]. Treatment typically involves metronidazole or vancomycin; in many cases, continuation of rifampin-based TB therapy is feasible, contingent on the patient’s overall clinical status and TB severity [86,87]. Effective treatment of TB coinfections involves coordinating antimycobacterial therapy with targeted antimicrobial regimens, factoring in the potential for drug interactions and toxicities. In patients with *S. aureus* and TB coinfection, both antibiotic and antituberculosis therapies must be tailored to address multidrug-resistant strains and comorbid conditions [73]. Novel research into multiepitopic vaccines targeting *Klebsiella pneumoniae* and *Mycobacterium tuberculosis* holds promise for proactive disease prevention [88]. As bacterial coinfections can significantly elevate morbidity and mortality, ongoing research focuses on vaccine development, advanced diagnostics, and improved management protocols [74]. By integrating molecular diagnostics, enhancing antibiotic stewardship, and refining combination therapies, clinicians can better address TB coinfections ranging from *S. pneumoniae* and *S. aureus* to *Pseudomonas aeruginosa* and *C. difficile* and ultimately improve patient outcomes in high-burden settings [15,89].

In LMICs, the management of tuberculosis and bacterial coinfections is further complicated by factors such as delayed diagnosis, inadequate treatment, and high rates of antibiotic resistance [66,68,69]. These challenges contribute to increased morbidity and mortality, underscoring the need for improved diagnostic capabilities, more robust infection control practices, and the development of novel antibiotics or treatment regimens to combat resistant strains [74]. Furthermore, addressing underlying conditions like malnutrition and HIV, which are prevalent in LMICs, is essential for lowering both the incidence and severity of coinfections [69].

## 4. Fungal Coinfections

Pulmonary tuberculosis (TB) and fungal coinfections pose a substantial challenge in clinical settings, largely due to overlapping symptoms that complicate diagnosis and the need for complex, simultaneous management. These challenges are particularly pronounced in LMICs, where limited resources often impede timely and accurate diagnosis, as well as access to effective treatment regimens. Research indicates that fungal coinfections are notably prevalent among patients with pulmonary TB in these regions, with *Aspergillus* and *Candida* species being the most common pathogens involved [90,91]. This co-occurrence can lead to diagnostic delays, intensify treatment complexities, and ultimately worsen patient outcomes, thereby highlighting an urgent need for more effective diagnostic tools and targeted therapeutic strategies in LMICs [90]. The prevalence of *Aspergillus* coinfection in TB patients in Asia and Africa is approximately 15.4%, with *Aspergillus fumigatus* being the most frequent species [92]. In Indonesia, a study found that 32% of multidrug-resistant TB (MDR-TB) patients had positive serology for *Aspergillus*, indicating a significant burden of coinfection [90,91]. *Candida* coinfection is even more prevalent, with a pooled prevalence of 25.7% globally, and *Candida albicans* being the most common species [93,94]. Coinfections are more common in older adults, particularly those over 40 years of age [92]. Several risk factors contribute to the susceptibility of TB patients to fungal coinfections [91]. These include advanced age, diabetes, smoking, and a low body mass index, which are common among TB patients and exacerbate their vulnerability to fungal infections [93]. The use of prolonged antibiotics and immunosuppressive agents also increases the risk of fungal infections in these patients [92].

Fungal coinfections in TB patients often complicate the clinical picture due to overlapping clinical and radiological features shared by TB and fungal infections, notably *Aspergillus* species and *Candida* [91,94]. These similarities manifesting as fever, cough, and hemoptysis can lead to misdiagnosis, including misinterpretation as TB reactivation, which delays appropriate treatment and worsens patient outcomes [95]. Furthermore, invasive aspergillosis may present in both pulmonary and central nervous system forms, adding to the complexity of clinical assessment and management [90]. The presence of fungal pathogens can also complicate the treatment of TB, as these infections may not respond to standard antitubercular drugs, necessitating the use of antifungal treatments like itraconazole and amphotericin B [96]. Pulmonary tuberculosis and fungal coinfections pose a substantial challenge in clinical settings, particularly in LMICs where resource constraints hamper timely diagnosis and treatment. The diagnosis of TB and *Aspergillus* coinfections in these resource-poor settings is further hindered by limited access to rapid and accurate laboratory diagnostics, often resulting in diagnostic delays [97]. Mycological and bacteriological investigations are recommended to identify secondary infections and tailor treatment plans accordingly [98]. Advanced imaging and serological tests, such as the detection of *Aspergillus* IgG antibodies, are essential for accurate diagnosis [90]. Accurate diagnosis of Candida coinfection in TB patients is critical. Techniques such as culture on Sabouraud Dextrose Agar and identification through Gram staining are used to detect *Candida* species in sputum samples [96]. Implementing appropriate diagnostic measures can help in the timely management of these coinfections, reducing the risk of chronicity and improving the overall prognosis for TB patients [94,97].

Managing TB and *Aspergillus* coinfections poses a significant challenge due to potential drug interactions particularly between rifampicin and antifungal agents like voriconazole [91,95]. As a result, careful selection and close monitoring of treatment regimens are essential. Typically, therapy involves a combination of antifungal medications and anti-TB drugs, and in some cases, surgical intervention may be required (in the presence of aspergillomas). Effective management of *Candida* coinfection similarly depends on antifungal therapy most commonly itraconazole or amphotericin B administered alongside standard antitubercular therapy [96,99]. Addressing TB and fungal coinfections in LMICs requires a comprehensive strategy that not only enhances laboratory capacity, improves access to diagnostics, and integrates effective treatment regimens, but also acknowledges and addresses socioeconomic and cultural barriers that influence treatment adherence and outcomes [97,100].

## 5. Parasitic Coinfections

Tuberculosis and parasitic coinfections pose significant public health challenges, particularly in LMICs where these diseases are endemic and frequently coexist in tropical and subtropical regions with high burdens of both, often overlapping geographically [101].

In the context of parasitic coinfections, we have specifically focused on helminth infections and intestinal parasites, as these represent the most prevalent parasitic conditions among TB patients, particularly in LMICs [101,102].

Socio-demographic factors such as gender, age, HIV status, and migration from co-endemic areas further heighten susceptibility to coinfections [4]. In Ethiopia, the pooled prevalence of intestinal parasitic coinfection among TB patients is estimated at 33%, with *Ascaris lumbricoides*, *Hookworm* (*Necator americanus* and *Ancylostoma duodenale*), *Giardia lamblia*, and *Strongyloides stercoralis* being most common [102]. In Uganda, a study among drug-resistant TB patients reported a lower but still notable 4.7% prevalence of helminth coinfection [103]. In Malaysian aborigines, parasitic infections were highly prevalent among TB patients 100% compared to 94.6% in healthy controls with toxoplasmosis in particular showing a positive association with active pulmonary TB. Conversely, trichuriasis was associated with a reduced likelihood of active TB, indicating that different parasites may have varying effects on TB susceptibility [104]. Similarly, in Burkina Faso, a high frequency of parasitic coinfections has been documented, with protozoal infections significantly more common in TB patients compared to non-TB individuals [105]. Helminth infections are particularly prevalent in regions with high TB incidence, and their impact on TB can be significant. By inducing a Th2-type immune response, helminths may impair the Th1 response essential for controlling *Mycobacterium tuberculosis*, thereby exacerbating TB severity [106,107]. This immunological interplay underscores the importance of identifying and treating parasitic coinfections in TB-endemic areas to improve overall patient outcomes. Additionally, intestinal parasites may shift the immune response from a cell-mediated (Th1) to a humoral (Th2) focus, suppressing the host’s capacity to control TB effectively [106]. Coinfections can exacerbate TB severity by increasing lung inflammation and accelerating disease progression; for instance, helminth-induced arginase-1 expression in macrophages can worsen pulmonary inflammation in TB patients [107]. Parasitic infections may also influence the pharmacokinetics of TB drugs, potentially leading to suboptimal treatment outcomes [6]. Moreover, coinfections can reduce the efficacy of TB vaccines such as the Bacillus–Calmette–Guérin (BCG) vaccine by modulating the host’s immune response [108].

Effective management of helminth-TB coinfections requires a combination of strategies. Mass drug administration of antihelminthic drugs, combined with TB treatment and vaccination, has been shown to be effective. Educational campaigns and sanitation improvements are also crucial components of a comprehensive control strategy [109]. Annual short-burst anthelmintic administration can reduce TB severity, although it may not significantly impact TB prevalence [110].

Helminth infections can lead to immunological changes such as reduced CD4 T cell counts and altered monocyte activation, which can impair TB control. These changes are often reversible with antihelminthic treatment, highlighting the importance of addressing helminth infections in TB patients [111].

Despite the recognized clinical impact of TB and parasitic coinfections, more research is needed to elucidate the precise immunological and pharmacological mechanisms underlying these interactions [112]. Developing integrated treatment strategies that target both TB and parasitic infections could significantly improve patient outcomes in co-endemic regions [101]. Finally, there is a pressing need for enhanced diagnostic tools and treatment protocols that account for coinfections, particularly in LMICs where the burden of these diseases is highest [61,101].

## 6. The Impact of Socioeconomic Factors on Coinfection Rates in LMICs

**Figure 2:**
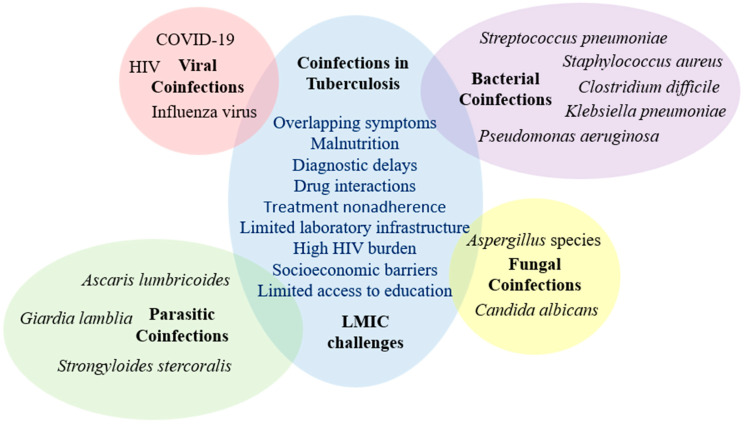
Coinfections in tuberculosis and LMICs income challenges (LMIC).

The impact of socioeconomic factors on coinfection rates in tuberculosis is a critical area of study, particularly in low- and middle-income countries. Socioeconomic status (SES) significantly influences the prevalence and outcomes of TB and its associated coinfections [1]. Individuals with lower SES often face barriers such as limited access to healthcare, inadequate nutrition, and poor living conditions, which can exacerbate the risk and severity of coinfections [31,54]. Limited access to healthcare is a major factor contributing to higher coinfection rates among individuals with low SES. In many LMICs, healthcare infrastructure is often under-resourced, leading to delayed diagnosis and treatment of TB and its coinfections [3,25]. For instance, the lack of comprehensive diagnostic facilities can result in underdiagnosis or misdiagnosis of coinfections, such as bacterial pneumonia caused by *Streptococcus pneumoniae* or *Staphylococcus aureus* [64,65]. This can lead to prolonged illness and increased transmission rates within communities. Inadequate nutrition is another significant factor (Figure 2).

Malnutrition weakens the immune system, making individuals more susceptible to infections. This is particularly concerning for TB patients, as a compromised immune system can facilitate the progression of coinfections. Studies have shown that malnutrition is prevalent among TB patients in LMICs, further complicating their treatment and recovery [69]. Additionally, malnutrition can impair the efficacy of TB treatment, leading to poorer outcomes and higher mortality rates. Poor living conditions, including overcrowded housing and lack of sanitation, also contribute to the spread of TB and its coinfections. Overcrowded living conditions facilitate the transmission of airborne pathogens, such as the influenza virus, which can coinfect TB patients and worsen their clinical outcomes [23]. Moreover, inadequate sanitation can lead to the spread of parasitic infections, such as helminths, which can further complicate TB management [102]. The psychological impact of living in poverty should not be underestimated. The stress associated with low SES can lead to mental health issues, which have been shown to negatively impact TB treatment adherence and outcomes. For example, anxiety and depression are common among TB patients and can be exacerbated by coinfections, such as those involving COVID-19 [49,50]. This highlights the need for integrated healthcare approaches that address both physical and mental health needs of TB patients in LMICs.

Beyond traditional socioeconomic determinants, global challenges such as climate change, migration, and conflict are increasingly shaping TB coinfection dynamics in LMICs. The intersection of TB and climate change presents significant challenges for LMICs. Climate change impacts TB through various pathways, influencing both the prevalence and management of the disease. Climatic factors such as changes in temperature, humidity, and precipitation can affect TB transmission dynamics; for example, higher temperatures have been associated with increased TB cases, while increased humidity and rainfall may reduce transmission [113,114]. In addition, extreme weather events can lead to population displacement, thereby increasing the number of vulnerable individuals susceptible to TB. Moreover, climate change can disrupt health systems by interfering with TB diagnostic and treatment services, further complicating disease management in resource-limited settings [114].

Furthermore, socioeconomic disparities often result in limited access to education, which can affect individuals’ awareness and understanding of TB and its coinfections. Lack of knowledge about disease prevention and management can lead to delayed healthcare seeking behavior and poor adherence to treatment regimens. This is particularly problematic in regions with high HIV prevalence, where coinfection with TB is common and requires complex treatment strategies. In summary, socioeconomic factors play a crucial role in the epidemiology of TB coinfections. Addressing these factors through improved healthcare access, better nutrition, enhanced living conditions, and comprehensive education programs is essential for reducing the burden of TB and its associated coinfections in LMICs [97,100]. Efforts to mitigate these socioeconomic disparities can lead to better health outcomes and reduced transmission rates, ultimately contributing to the global fight against TB [1,3].

## 7. TB Coinfection Management Models

TB coinfections with other pathogens presents a significant public health challenge, particularly in resource-limited settings. Effective management models are essential to improve treatment outcomes and reduce mortality rates.

Decentralization of TB treatment to primary health clinics (PHCs) has shown promising results in rural South Africa. Patients with TB/HIV coinfection who were down-referred from district hospitals to PHCs experienced higher treatment success rates and lower mortality compared to those who remained at the hospital. However, there was a higher default rate among down-referred patients, primarily due to failed linkage of care during the transfer process. This highlights the need for improved patient education, active communication between hospitals and PHCs, and better tracing of patients lost to follow-up [115]. Mathematical models have been developed to optimize the management of TB and HIV coinfection by integrating various control measures into a unified framework. These models combine treatment strategies such as antiretroviral therapy for HIV and specific regimens for both latent and active TB, enabling a comprehensive approach to disease control. Optimal control theory has been applied to derive strategies that reduce the number of individuals with active TB and AIDS. Simulation results suggest that implementing multiple control measures concurrently can significantly reduce the prevalence of coinfection [116]. The use of such models is particularly valuable in resource-limited settings, where optimizing the impact of each intervention is crucial for reducing morbidity and mortality associated with TB/HIV coinfection [117].

Research using macaque models has identified immune mechanisms that can suppress the reactivation of latent TB in the context of HIV coinfection. CD8+ T cells and B cells play a crucial role in controlling TB, even in the absence of CD4+ T cells. These findings have implications for developing prophylactic and therapeutic measures against TB and HIV [89]. Additionally, BCG vaccination in macaque models has shown potential in controlling TB-induced disease and reducing viral load, supporting its use in evaluating interventions against TB reactivation [118].

Despite the success of decentralized and integrated care models, challenges remain in ensuring effective linkage of care and preventing treatment default. Future research should focus on enhancing communication between healthcare facilities and developing strategies to identify and support potential defaulters. Additionally, further studies are needed to explore the role of immune mechanisms in TB control and to develop vaccines and immunotherapies that leverage these insights [89,115].

## 8. Conclusions

TB coinfections pose a multifaceted challenge in LMICs, where constrained resources, high HIV prevalence, and socioeconomic barriers complicate the clinical management and control of TB. Viral coinfections such as HIV, SARS-CoV-2, and influenza amplify the risk of TB reactivation and progression, while bacterial, fungal, and parasitic coinfections further complicate diagnosis and treatment. Overlapping clinical features and drug interactions, particularly in HIV–TB-coinfected patients, underscore the critical need for comprehensive and integrated diagnostic and therapeutic strategies. Strengthening laboratory capacities, implementing targeted antibiotic stewardship, and addressing underlying socioeconomic determinants including malnutrition, overcrowding, and limited healthcare access are pivotal steps toward mitigating the burden of TB coinfections. Future research should focus on developing rapid diagnostic tools, optimizing multidrug regimens, and formulating public health policies that integrate TB care with broader infectious disease control measures. Collectively, these efforts are essential for improving patient outcomes and curbing the global impact of TB in LMICs.

## Data Availability

The data presented in this study are available upon request from the corresponding author.
